# Dismantling the Taboo against Vaccines in Pregnancy

**DOI:** 10.3390/ijms17060894

**Published:** 2016-06-07

**Authors:** Maurizio de Martino

**Affiliations:** Department of Health Sciences, University of Florence, Anna Meyer Children’s University Hospital, viale Gaetano Pieraccini 24, Florence 50139, Italy; maurizio.demartino@unifi.it; Tel.: +39-055-566-2494; Fax: +39-055-422-1012

**Keywords:** vaccine, immunization, pregnancy, pertussis, influenza, tetanus

## Abstract

Vaccinating pregnant women in order to protect them, the fetus, and the child has become universal in no way at all. Prejudice in health professionals add to fears of women and their families. Both these feelings are not supported by even the smallest scientific data. Harmlessness for the mother and the child has been observed for seasonal, pandemic, or quadrivalent influenza, mono, combined polysaccharide or conjugated meningococcal or pneumococcal, tetanus toxoid, acellular pertussis, human papillomavirus, cholera, hepatitis A, Japanese encephalitis, rabies, anthrax, smallpox, yellow fever, mumps, measles and rubella combined, typhoid fever, inactivated or attenuated polio vaccines, and *Bacillus Calmétte Guerin* vaccines. Instead, the beneficial effects of influenza vaccine for the mother and the child as well as of pertussis vaccine for the child have been demonstrated. Obstetrician-gynecologists, general practitioners, and midwives must incorporate vaccination into their standard clinical care. Strong communication strategies effective at reducing parental vaccine hesitancy and approval of regulatory agencies for use of vaccines during pregnancy are needed. It must be clear that the lack of pre-licensure studies in pregnant women and, consequently, the lack of a statement about the use of the vaccine in pregnant women does not preclude its use in pregnancy.

## 1. Introduction

It is time to clear the hesitancy (at the very best) if not the horror (in the worst case) arousing in laymen, but regrettably also in many health professionals, when immunizations are proposed to pregnant women. Factors influencing vaccination acceptance have been excellently located [1]. Safety, need, effectiveness, or conflicting advice influence the laymen. Inadequate training, inadequate reimbursement, and increased workload influence health professionals.

A mixture of defensive medicine, the ancient visceral distrust against any artificial interference in pregnancy, and the current increasing mistrust against vaccines in the industrialized world may contribute to the proscription.

By contrast, we must be now convinced that these erroneous concepts and deriving harmful attitudes are exclusively based on mental laziness and low knowledge.

The protective effect in infants of maternal immunization during pregnancy was observed as early as 1879 when infants of mothers who had received the Jennerian vaccine during pregnancy resulted in their protection against smallpox [2].

## 2. Safety of Vaccines in Pregnancy

Since 1879 a large body of evidence, acquired in hundreds of thousands of cases, has accumulated on the safety of immunization in pregnancy using non live vaccines [3,4].

Studies in non-live vaccines include inactivated seasonal or pandemic influenza, mono- or combined polysaccharide or conjugated meningococcal, tetanus toxoid, acellular pertussis, human papillomavirus, cholera, hepatitis A, Japanese encephalitis, rabies vaccines, and anthrax vaccines [5,6,7,8,9,10,11,12,13,14,15,16,17,18,19,20,21,22,23,24,25].

Pregnant women are rightly excluded from prelicensure clinical trials of live-virus vaccines because of the theoretical risk of transmission of the vaccine virus to the fetus. Thus data do not exist deriving from prospective studies. As a consequence, as prudently as properly, live-virus vaccines are contraindicated for pregnant women. It is recommended to avoid immunization during pregnancy and to avoid pregnancy in the immediate period after administration of such vaccines [26,27,28].

However, there is a large body of evidence derived from retrospective studies based on women inadvertently vaccinated individually and included in special registers (such as the Vaccine Adverse Event Reporting System or the Varicella-Zoster Virus-Containing Vaccines Pregnancy Registry) or women vaccinated during mass vaccination campaigns or military women vaccinated due to the risk of biological warfare. Data indicate the absolute absence of side effects on pregnancy, mother, and child. Confidence is so strong that advisory agencies suggest that live vaccines “*must be considered basing on risk factors or special circumstances* and that *vaccination is not ordinarily an indication to terminate the* *pregnancy*” [26,27,28].

Safety has been demonstrated for smallpox, yellow fever (excluding a few reports of infection during breast feeding), mumps, measles and rubella combined vaccine, quadrivalent influenza, typhoid fever vaccine, oral attenuated poliomyelitis virus live vaccines, and Bacillus Calmétte Guerin [3,15,29,30,31,32,33,34,35,36]. There are some reports indicating that that vaccination with yellow fever vaccine may induce infection with the vaccine strain through breast milk.

As a matter of fact, the Varicella-Zoster Virus-Containing Vaccines Pregnancy Registry was closed after about 920 reports had been included of women who inadvertently received the varicella vaccine within three months before pregnancy or at any time during pregnancy. No effect on pregnancy, mother, or child was reported [37].

Several tens of thousands of pregnant women received the rubella vaccine in Argentina, Brazil, Costa Rica, Ecuador, El Salvador, and Paraguay during mass campaigns for rubella and congenital rubella syndrome elimination. No side effects were observed on pregnancies, mothers, or children. One baby showed at birth IgM antibodies against the vaccine virus, but no evidence of congenital rubella was found. This may indicate that the baby was “vaccinated” rather than “infected” during pregnancy [38,39,40].

Similar reassuring results were obtained on a total of 680 susceptible pregnant women who had received a rubella vaccine during pregnancy in the United States, West Germany, Sweden, and the United Kingdom: no infants with congenital rubella syndrome were born [41]. Some babies were born with IgM against the rubella virus, but with no sign of congenital rubella.

Findings concerning the safety of live vaccines in no way should be considered an incentive to deliberately administer these vaccines to pregnant women and we must continue to advise women to avoid pregnancy for one month after receiving the rubella-containing vaccine. It may be that this is an example of excessive caution, but it is wise in medical practice *primum non nocere*, (that is *first*, *do no harm*) is this sentence the brainchild of Hippocrates or Galen or Sydenham? It is still a contentious matter [42], but in any case the sentence is a pearl of wisdom.

On the other hand, two considerations deserve to be made. First: the safety of live vaccines in pregnancy is a convincing model of overall safety of immunization in pregnancy; second: these findings indicate that if a pregnant woman is inadvertently vaccinated, she should be counseled about the theoretical basis of concern for the fetus, but vaccination during pregnancy should not inevitably be a reason to consider termination of pregnancy.

## 3. Efficacy and Need

Several studies indicate that immunization during pregnancy with tetanus, diphtheria and acellular pertussis vaccine is as immunogenic as in non-pregnant women [16,24,26,43]. Pregnant women mount a lively humoral response to tetanus and diphtheria toxoids as well as to *Bordetella pertussis* antigens (pertussis toxin, filamentous hemaglutinin, and pertactin). By contrast, proliferative and interferon-γ responses (expression of cellular immunity) are transient and impaired [43].

The Centers for Disease Control and Prevention, as well as the American Academy of Pediatrics, recommend that pregnant women must be immunized against tetanus, influenza, and pertussis [12,44].

Maternal vaccination against tetanus is crucial to avoid *tetanus neonatorum*. Studies carried out in the sixties had shown that the vaccine reset the mortality rate from 7.8 to 0/100 live births [45]. In the late 1980s these findings induced the World Health Organization (WHO) to undertake the WHO’s *Maternal and Neonatal Tetanus Elimination Initiative* which led to a 98% decrease of deaths caused by *tetanus neonatorum* [20].

Fever in pregnancy *per se* predisposes for an increased risk for cleft palate, and heart or neural tube defects [46]. During pregnancy women display a reduced response of CD8 lymphocytes and dendritic cells to influenza virus at a time in which physiological hemodinamic and respiratory changes are also occurring [5,47]. This convergence of harmful mechanisms causes an increased risk of developing an influenza infection and the particularly severe influenza disease [5,47]. During the 2009 H1N1 pandemic influenza infected pregnant women had a four times increased risk of hospitalization as compared to non-pregnant women [48,49]. Twelve percent of pregnancy-related deaths were attributed to confirmed or possible influenza infection [48,49]. These phenomena occurred also during the 1918–1919 Spanish, 1956 Asian, and 2010 H1N1 pandemics [50,51,52].

Influenza in pregnancy is dangerous not only for the mother but also for the child. A meta-analysis carried out on 33 studies, which included more than 1600 children exposed *in utero* to maternal influenza, showed that about 3.5% of the children manifested neural tube, limbs, heart or digestive defects, cleft palate, or hydrocephalus.

Since an efficient transplacental passage of antibodies against the influenza virus does occur, the child is protected (efficacy: 68%) from the disease during the first semester of life [5]. This is the age range when the vaccine is not recommended, but it is also the age range when the risk of death is higher as compared to other ages [5].

Despite pertussis vaccination, morbidity and mortality due to *Bordetella pertussis* remains high worldwide, particularly among newborns and infants aged less than three months [53,54,55]. More than 90% of fatalities occur in this age range. At least two immunization strategies can be imagined to protect the infant.

The first one is the cocooning strategy which involves parents, caregivers, and others in close proximity. The rational of this strategy is that if they are protected from pertussis they indirectly protect infants from transmission. The main obstacles in the cocooning strategy are the need to vaccinate multiple households, and the acceptance of vaccination by all the loose cannon of non-household sources of infection [56,57]. Far from unimportant is the concern that acellular pertussis vaccines, which behave differently from natural infection and the whole cell vaccine, prevent severe disease but not colonization and transmission. This may depend on a different T-cell response: natural infection and whole cell vaccine stimulate a robust *Bordetella pertussis*-specific T_H_17 memory cell response (crucial against intracellular infections) whereas acellular vaccines induce a T_H_1/T_H_2 response. Thus, the cocoon strategy may fail to prevent colonization of family members and transmission to the newborn because *Bordetella pertussis* is transmitted to the infant by vaccinated but infected, even though asymptomatic, family members [58]. In addition, the timing of changes in age-specific attack rates and the phylodynamic analysis of sequences are consistent with the significant role of asymptomatic transmission in pertussis epidemiology [59]. In summary, the failure of programs aimed to reduce infant pertussis by applying the cocoon strategy through the use of the acellular vaccine is not surprising [60].

The second strategy is vaccinating pregnant women, thereby directly conferring protection through the passive transfer of pertussis antibodies. The efficacy and effectiveness of this approach in preventing pertussis [61] and the placental transfer of maternal antibodies [62] have been demonstrated. At least three problems must be overcome [13,62,63,64,65]: (a) the lower efficacy in preterm infants who acquire a lower dosage of maternal antibodies; (b) the lower antibody response to the first dose of pertussis vaccine (even though the booster has been shown to elicit a response similar to that of infants born to non-immunized women); (c) the mutual interference between passively acquired maternal antibodies and antibodies actively elicited through vaccination [66]. Interference manifests *in vitro* as well as *in vivo* not as the amount of antibodies as much as the quality of antibodies and occurs only when the same vaccine is used in the mother and in the infant. Thus the solution is likely immunizing the mother and the infant with two different vaccines. There is another contrasting theory stating that vaccinating with the same vaccine would be beneficial, since immune memory would play a role on later antibody responses and antibody affinity (the so-called genetic imprinting).

Finally, two questions [10]: (a) should vaccinations be administered routinely to all pregnant women (as a consequence it becomes the first dose for the infant) or reserved in the case of outbreak? (b) should vaccinations be administered at every pregnancy regardless of the interval between pregnancies?

The cost-effectiveness of maternal vaccination *vs.* the cocooning strategy has been evaluated [67]: the vaccine in pregnancy is more cost-effective, and decreases the number of cases of pertussis in infants by 33% (as compared to 20% with the cocooning strategy), hospitalizations by 38% (compared to 19% with the cocooning strategy), and deaths by 49% (as compared to 16% with the cocooning strategy). The cost of vaccinating the woman during pregnancy is about one third of the cost of cocooning (which comes very close to $1200).

## 4. Conclusions

What there is to do? Not a little:

1. a Copernican revolution must be carried out in our vision of vaccination during pregnancy [68,69]. Obstetrician-gynecologists, general practitioners, and midwives, who already provide a large and valuable set of medical care to women, must incorporate vaccination into their standard clinical care. They must be convinced that vaccination in pregnant women has now become an ineluctable preventive measure which protects the mother, the fetus, and the baby; recommendations of healthcare providers are the keystone which induces vaccine uptake;
2. in spite of the fact that physicians have a major influence on parental vaccine decision, physician-targeted communication intervention does not reduce maternal vaccine hesitancy or improve physician self-efficacy. Challenges for health care providers in informing and educating pregnant women range from fear of needles, lack of knowledge about the vaccine, lack of perceived benefits, and lack of knowledge about the severe consequences of the preventable disease. Research is needed to identify other communication strategies that are effective at reducing parental vaccine hesitancy in the primary care setting [70]. New methods to fully capture the benefits of vaccination and increase vaccine acceptance are two key elements that all stakeholders involved in vaccine policy, including scientists, must consider very carefully [71];
3. evidentiary balance must replace the balanced at par description of opposing theories (in our case; vaccination *vs.* non-vaccination in pregnancy) where a preponderance of evidence points to a particular conclusion [72,73];
4. regulatory issues must receive more attention [74]. In many countries vaccines are not contraindicated for use in pregnant women, but no vaccine is licensed for use specifically during pregnancy. Approval of regulatory agencies for therapeutic goods for use of vaccines during pregnancy, in order to prevent disease in the mother and/or infant, would have weighty consequences on uptake and usage in pregnant women resulting in labeling which will assist physicians in facilitating the use of the vaccine during pregnancy. It should be stressed that “*the lack of pre-licensure studies in pregnant women and consequently the lack of a statement about the use of the vaccine in pregnant women does not preclude its use in pregnancy. Such use is not off label and recommendations of immunization technical advisory groups are not inconsistent with regulatory agencies labelling*” [74]. Doing nothing is a risk [75].

## References

[1] Wilson R.J., Paterson P., Jarrett C., Larson H.J. (2015). Understanding influencing vaccination acceptance during pregnancy globally: A literature review. Vaccine.

[2] Burckhardt A.E. (1879). On intrauterine vaccination. Dtsch. Arch. Win. Med..

[3] Keller-Stanislawsky B.K., Englund J.A., Kang G., Mangtani P., Neuzil K., Nohynek H., Pless R., Lambach P., Zuber P. (2014). Safety immunization during pregnancy: A review of the evidence of selected inactivated and live attenuated vaccines. Vaccine.

[4] Naleway A.L., Kurosky S., Henninger M.L., Gold R., Nordin J.D., Kaìharbanda E.O., Irving S., Cheetham C., Nakasato C., Glanz J.M. (2014). Vaccinations given during pregnancy, 2002–2009. A descriptive study. Am. J. Prev. Med..

[5] Tamma P.D., Ault K.A., del Rio C., Steinhoff M.C., Halsey N., Omer S.B. (2009). Safety of influenza vaccination during pregnancy. Am. J. Obs. Gynecol..

[6] Zheteyeva Y.A., Moro P.L., Tepper N.K., Rasmussen S.A., Barash F.E., Revzina N.V., Kissin D., Levis P.W., Yue X., Haber P. (2012). Adverse event reports after tetanus toxoid, reduced diphteria toxoid, and acellular pertussis vaccines in pregnant women. Am. J. Obstet. Gynecol..

[7] Hashim R., Khatib A.M., Enwere G., Park J.K., Reyburn R., Ali M., Chang N.Y., Kim D.R., Ley B., Thriemer K. (2012). Safety of the recombinant cholera toxin B subunit, killed whole-cell (rBS-WC) oral cholera vaccine in pregnancy. PLoS Negl. Trop. Dis..

[8] Anselem O., Parat S., Théau A., Floret D., Tsatsaris V., Goffinet F., Launay O. (2014). Vaccination and pregnancy. Presse Med..

[9] Loubet P., Kerneis S., Anselem O., Tsatsaris V., Goffinet F., Launay O. (2014). Should expectant mothers be vaccinated against flu? A safety review. Expert Opin. Drug Saf..

[10] McIntyre P.B., Clark T.A. (2014). Pertussis vaccine in pregnancy—First dose for every infant?. Lancet.

[11] Lindsey B., Kampmann B., Jones C. (2013). Maternal immunization as a strategy to decrease susceptibility to infection in newborn infants. Curr. Opin. Infect. Dis..

[12] Committee of Infectious Disease (2015). Recommendations for prevention and control of influenza in children. Pediatrics.

[13] Healy C.M. (2012). Vaccines in pregnant women and research initiatives. Clin. Obst. Gyncol..

[14] Kharbanda E.O., Velasquez-Benitez G., Lipkind H.S., Klein N.P., Cheetham C., Naleway A., Omer S.B., Hambidge S.J., Lee G.M., Jackson M.L. (2014). Evaluation of the association of maternal pertussis vaccination with obstetric events and birth outcomes. JAMA.

[15] Pellegrini C., McCabe E.R.B. (2015). Maternal immunization at crossroads. Vaccine.

[16] Munoz F.M., Bond N.H., Maccato M., Pinell P., Hammill H.A., Swamy G.K., Walter E.B., Jackon L.A., Englund J.A., Edwards M.S. (2014). Safety and immunogenicity of tetanus diphteria and acellular pertussis (Tdap) immunization during pregnancy in mothers and infants: A randomized clinical study. JAMA.

[17] Larson H.J. (2015). Maternal immunizartion: The new “normal” (or it should be). Vaccine.

[18] Moro P.L., McNeil M.M., Sukumaran L., Broder K.R. (2015). The Centers for Disease Control and Prevention’s public health response to monitoring Tdap safety in pregnant women in the United States. Hum. Vaccines Immunother..

[19] Sukumaran L., McCarthy N.L., Kharbanda E.O., Weintraub E.S., Vasquez-Benitez G., McNeil M., Rongxia L., Klein N.P., Hambridge S.J., Naleway A.L. (2015). Safety of tetanus toxoid, reduced diphteria toxoid, and acellular pertussis and influenza vaccinations in pregnancy. Obstet. Gynecol..

[20] World Health Organization. http:/www.who.int/immunization_monitoring/disease/MNT.initiative/en/.

[21] Li R., Klein N.P., Hambridge S.J., Naleway A.L., Lugg M.M., Li R., Weintraub E.S., Bednarczyk R.A., King J.P., DeStefano F. (2015). Association of Tdap vaccination with acute events and adverse birth outcomes among pregnant women with prior tetanus-containing immunizations. JAMA.

[22] Colin A.M., Bukowinski A.T., Gumbs G.R. (2015). Analysis of pregnancy and infant health outcomes among women in the National Smallpox Vaccine in Pregnancy Registry who received Anthrax Vaccine Adsorbed. Vaccine.

[23] (2015). HPV vaccines and pregnancy. Prescrire Int..

[24] Maertens K., Caboré R.N., Hygen K., Hens N., van Damme P., Leuridan E. (2016). Pertussis vaccination during pregnancy in Belgium: Results of a prospective controlled cohort study. Vaccine.

[25] Hoang H.T.T., Leuridan E., Maertens K., Nguyen T.D., Hens N., Vu N.H., Caboré R.N., Duong H.T., Huygen K., van Damme P. (2016). Pertussis vaccination during pregnancy in Vietnam: Results of a randomized controlled trial pertussis vaccination during pregnancy. Vaccine.

[26] Centers for Disease Control and Prevention Guidelines for Vaccinating Pregnant Women. http://www.cdc.gov/vaccines/pubs/preg-guide.htm#ppsv23.

[27] Centers for Disease Control and Prevention (2006). General recommendations on immunization: Recommendations of the Advisory Committee on Immunization Practices (ACIP). MMWR Recomm. Rep..

[28] Swamy G.K., Heine R.P. (2015). Vaccinations for pregnant women. Obstet. Gynecol..

[29] Centers for Disease Control and Prevention (1996). The role of BCG vaccine in the prevention and control of tuberculosis in the United States. A joint statement by the Advisory Council for the Elimination of Tuberculosis and the Advisory Committee on Immunization Practices. MMWR Recomm. Rep..

[30] Ryan M.A., Gumbs G.R., Conlin A.M., Sevick C.J., Jacobson I.G., Snell K.J., Spooner C.N., Smith T.C. (2008). Evaluation of preterm births and birth defects in live born infants of US military women who received smallpox vaccine. Birth Defects Res. Clin. Mol. Teratol..

[31] Suzano C.E., Amaral E., Sato H.K., Papaiordanau P.M., Campinas Group on Yellow Fever Immunization during Pregnancy (2006). The effects of yellow fever immunization (17DD) inadvertently used in early pregnancy during a mass campaign in Brazil. Vaccine.

[32] Thomas R.E., Lorenzetti D.L., Spragins W., Jackson D., Williamson T. (2012). The safety of yellow fever vaccine 17D or 17DD in children, pregnant women, HIV+ individuals, and older persons: Systematic review. Am. J. Trop. Med. Hyg..

[33] Kharbanda E.O., Vazquez-Benitez G., Lipkind H.S., Klein N.P., Cheethmam T.C., Naleway A.L., Lee G.M., Hambidge S., Kackson M.L., Omer S.B. (2016). Maternal Tdap vaccination: Coverage and acute safety outcomes in the vaccine safety datalink, 2007–2013. Vaccine.

[34] Rasmussen S.A., Watson A.K., Kennedy E.D., Broder K.R., Jamieson D.J. (2014). Vaccines and pregnancy: Past, present, and future. Semin. Fetal Neonatal Med..

[35] Haber P., Moro P.L., Cano M., Lewis P., Stewart B., Shimabukuro T.T. (2015). Post-licensure surveillance of quadrivalent live attenuated influenza vaccine United States, Vaccine Adverse Event Reporting System (VAERS), July 2013–June 2014. Vaccine.

[36] Sukumaran L., McNeil M.M., Moro P.L., Lewis P.W., Winiecki S.K., Shimabukuro T.T. (2015). Adverse Events Following Measles, Mumps, and Rubella Vaccine in Adults Reported to the Vaccine Adverse Event Reporting System (VAERS), 2003–2013. Clin. Infect. Dis..

[37] Marin M., Willis E.D., Marko A., Rasmussen S.A., Bialek S.R., Dana A. (2014). Closure of varicella-zoster virus-containing vaccines pregnancy registry—United States, 2013. MMWR Morb. Mortal. Wkly. Rep..

[38] Castillo-Solórzano C., Reef S.E., Morice A., Vascones N., Chevez A.E., Castalia-Soares R., Torres C., Vizzotti C., Ruiz Matus C. (2011). Rubella vaccination of unknowingly pregnant women during mass campaigns for rubella and congenital rubella syndrome elimination, the Americas 2001–2008. J. Infect. Dis..

[39] Soares R.C., Siqueira M.M., Toscano C.M., Maia M.L., Flannery B., Sato H.K., Will R.M., Rodrigues R.C., Oliveira I.C., Barbosa T.C. (2011). Follow-up study of unknowingly pregnant women vaccinated against rubella in Brazil, 2001–2002. J. Infect. Dis..

[40] Pardon F., Vilariño M., Barbero P., Garcia G., Outon E., Gil C., Vera A., Rossi S., Distefano A. (2011). Rubella vaccination of unknowingly pregnant women during 2006 mass campaign in Argentina. J. Infect. Dis..

[41] Tookey P.A., Jones G., Miller B.H., Peckham C.S. (1991). Rubella vaccination in pregnancy. CDR (Lond. Engl. Rev.).

[42] Smith C.M. (2005). Origin and uses of *primum non nocere*—Above all, do no harm!. J. Clin. Pharmacol..

[43] Huygen K., Caboré R.N., Maertens K., Van Damme P., Leuridan E. (2015). Humoral and cell mediated immune responses to a pertussis containing vaccine in pregnant and nonpregnant women. Vaccine.

[44] Morbidity and Mortality Weekly Report (2011). Updated recommendations for use of tetanus toxoid; reduced diphtheria toxoid and acellular pertussis vaccine (Tdap) in pregnant women and persons who have or anticipate having close contact with an infant aged <12 months—Advisory Committee on Immunization Practices (ACIP), 2011. MMWR Morb. Mortal. Wkly. Rep..

[45] Newell K.W., Duenas Lehmann A., LeBlanc D.R., Garces Osorio N. (1966). The use of toxoid for the prevention of tetanus *neonatorum*. Final report of a double-blind controlled field trial. Bull. WHO.

[46] Werenberg Dreier J., Nybo Andersen A.-M., Berg-Beckhoff G. (2014). Systematic review and meta-analyses: Fever in pregnancy and health impacts in the offspring. Pediatrics.

[47] Vanders R.L., Murphy V.E., Gibson P.G., Hansbro P.M., Wark P.A. (2015). CD8 T cells and dendritic cells: Key players in the attenuated maternal immune response to influenza infection. J. Reprod. Immunol..

[48] Callaghan W.M., Creanga A.A., Jamieson D.J. (2015). Pregnancy-related mortality resulting from influenza in the United States during the 2009–2010 pandemic. Obstet. Gynecol..

[49] Rasmussen S.A., Jamieson D.J. (2014). 2009 H1N1 influenza and pregnancy—5 years later. N. Engl. J. Med..

[50] Harris J.W. (1919). Influenza occurring in pregnancy. JAMA.

[51] Freeman D.W., Barno A. (1959). Deaths from Asian influenza associated with pregnancy. Am. J. Obstet. Gynecol..

[52] Jamieson D.J., Honein M.A., Rasmussen S.A., Williams J.L., Swerdlow D.L., Biggerstaff M.S., Lindstrom S., Louie J.K., Christ C.M., Bohm S.R. (2009). H1N1 2009 influenza virus infection during pregnancy in the USA. Lancet.

[53] Berti E., Venturini E., Galli L., de Martino M. (2014). Management and prevention of pertussis infection in neonates. Expert Rev. Anti Infect. Ther..

[54] Berti E., Chiappini E., Orlandini E., Galli L., de Martino M. (2014). Pertussis is still common in a highly vaccinated infant population. Acta Paediatr..

[55] Chiappini E., Stival A., Galli L., de Martino M. (2013). Pertussis re-emergence in the post-vaccination era. BMC Infect. Dis..

[56] Forsyth K., Plotkin S., Tan T., Wirsing von König C.H. (2015). Strategies to decrease pertussis transmission to infants. Pediatrics.

[57] Wiley K.E., Zuo Y., Macartney K.K., McIntyre P.B. (2013). Sources of pertussis infection in young infants: A review of key evidence informing targeting of the cocoon strategy. Vaccine.

[58] Warfel J.M., Zimmerman L.I., Merkel T.J. (2014). Acellular pertussis vaccines protect against disease but fail to prevent infection and transmission in a nonhuman primate model. Proc. Natl. Acad. Sci. USA.

[59] Althouse B.M., Scarpino S.M. (2015). Asymptomatic transmission and the resurgence of *Bordetella pertussis*. BMC Med..

[60] Healy C.M., Rench M.A., Wootton S.H., Castagnini A. (2015). Evaluation of the impact of a pertussis cocooning program on infant pertussis infection. Pediatr. Infect. Dis. J..

[61] World Health Organization Pertussis Vaccines: WHO Position Paper. Geneva 2010. http://www.who.int/entity/wer/2010/wer8540.pdf.

[62] Gall S.A., Myers J., Pichichero M. (2011). Maternal immunization with tetanus-diphtheria-pertussis vaccine: Effect on maternal and neonatal serum antibody levels. Am. J. Obstet. Gynecol..

[63] Van den Berg J.P., Westerbeek E.A., van der Klis F.R., Berbers G.A.A., van Elburg R.M. (2011). Transplacental transport of IgG antibodies to preterm infants: A review of the literature. Early Hum. Dev..

[64] Moniz M.H., Beigi R.H. (2014). Maternal immunization. Clinical experiences, challenges, and opportunities in vaccine acceptance. Hum. Vaccines Immunother..

[65] Hardy-Fairbanks A.J., Pan S.J., Decker M.D., Johnson D.R., Greenberg D.P., Kirkland K.-B., Talbot E.A., Bernstein H.H. (2013). Immune responses in infants whose mothers received Tdap vaccine during pregnancy. Pediatr. Infect. Dis. J..

[66] Feunou P.F., Milcareck N., Locht C. (2016). Reciprocal interference of maternal and infant immunization in protection against pertussis. Vaccine.

[67] Terranella A., Asay G.R., Messonnier M.L., Clark T.A., Liang J.L. (2013). Pregnancy dose Tdap and postpartum cocooning to prevent infant pertussis: A decision analysis. Pediatrics.

[68] Leader S., Perales P.J. (1995). Provision of primary-preventive health care services by obstetrician-gynecologists. Obstet. Gynecol..

[69] Beeler J.A., Lambach P., Fulton T.R., Narayanan D., Ortiz J.R., Omer S.B. (2016). A systematic review of ethical issues in vaccine studies involving pregnant women. Hum. Vaccin. Immunother.

[70] Henrikson N.B., Opel D.J., Grothaus L., Nelson J., Scrol A., Dunn J., Faubion T.Y., Roberts M., Markuse E.K., Grossman D.C. (2015). Physician communication training and parental vaccine hesitancy: A randomized trial. Pediatrics.

[71] De Gregorio E. (2015). The path forward. Vaccine.

[72] Clarke C.E., Dixon G.N., Holton A., McKeever B.W. (2014). Including “evidentiary balance” in news media coverage of vaccine risk. Health Commun..

[73] Dixon G., Clarke C. (2013). The effect of falsely balanced reporting of the autism-vaccine controversy on vaccine safety perceptions and behavioral intentions. Health Educ. Res..

[74] Roberts J.N., Gruber M.F. (2015). Regulatory considerations in the clinical development of vaccines indicated for use during pregnancy. Vaccine.

[75] Brent R.L. (2006). Risks and benefits of immunizing pregnant women: The risk of doing nothing. Reprod. Toxicol..

